# Etiology of early hearing loss in Brazilian children

**DOI:** 10.1016/j.bjorl.2021.02.012

**Published:** 2021-03-20

**Authors:** Marina Faistauer, Alice Lang Silva, Têmis Maria Félix, Liliane Todeschini de Souza, Renata Bohn, Sady Selaimen da Costa, Letícia Petersen Schmidt Rosito

**Affiliations:** a Universidade Federal do Rio Grande do Sul, Faculdade de Medicina, Porto Alegre, RS, Brazil; b Hospital de Clínicas de Porto Alegre, Departamento de Otorrinolaringologia e Cirurgia de Cabeça e Pescoço, Porto Alegre, RS, Brazil; c Universidade Federal do Rio Grande do Sul, Faculdade de Medicina, Departamento de Oftalmologia e Otorrinolaringologia, Porto Alegre, RS, Brazil; d Hospital de Clínicas de Porto Alegre, Departamento de Genética, Porto Alegre, RS, Brazil

**Keywords:** Infant, Prevalence, Connexin 26, Auditory neuropathy, Congenital

## Abstract

**Introduction:**

Hearing loss etiology depends on the population studied as well as on the ethnicity and the socio-economic condition of the analyzed region. Etiological diagnosis contributes to the improvement of preventive measures and to the early identification of this deficiency.

**Objective:**

To identify the etiological factors of hearing loss and its prevalence in a tertiary hospital in southern Brazil, to verify the frequency of mutations in GJB2 and GJB6 genes, and to correlate the degree of hearing loss with the etiological factors of deafness.

**Methods:**

This prevalence study involved 140 children with bilateral sensorineural or mixed hearing loss. Medical history, physical examination, audiometry, and evoked auditory brainstem response were conducted. Imaging and genetic examinations were also performed.

**Results:**

Etiologies and their prevalence were as follows: (a) indeterminate causes, 31.4%; (b) conditions related to neonatal period, 22.1%; (c) genetic, 22.1%; (d) auditory neuropathy, 10%; (e) other factors (cortical malformation, intracranial hemorrhage, and internal ear malformations), 7.9% and (f) congenital infections, 6.4%. Within the genetic cases, ten homozygous and seven heterozygotes of the 35delG mutation were identified, besides two cases of rare variants of *GJB2*: p.Try172* and p.Arg184Pro. One case with homozygosis of del(GJB6-D13S1830) was found. Regarding severity of hearing loss, in 78.6% of the cases the degree of hearing loss was profound and there were no significant differences when comparing between etiologies.

**Conclusion:**

The number of indeterminate etiologies is still high and congenital CMV infection may be a possible cause of undiagnosed etiology for hearing loss. The predominance of etiologies related to neonatal conditions and infectious causes are characteristic of developing countries. The most prevalent mutation was 35delG, the main GJB2 gene, probably because of the European influence in the genotype of our population.

## Introduction

Deafness is the most common congenital sensory deficiency, with an incidence of one to two cases per 1000 live births.^1^, ^2^, ^3^, ^4^, ^5^, ^6^, ^7^ This significant prevalence results in a major sociocultural impact since deafness significantly interferes with the processes of appropriation of oral and written language. The limitation of access to orality, on its own, requires numerous adaptations in the various social and family relations.^5^, ^7^, ^8^, ^9^

The main etiological factors of hearing loss have a variable prevalence since they are directly influenced by the socio-economic development, ethnicity, and region of a given country.^3^ In developed countries, most cases of hearing loss are genetic in nature (50%–60% of cases), which are comprised of the syndromic forms (15%–30%) and non-syndromic forms (70%–85%).^10^ From the remaining, approximately 35% are related to infectious diseases or secondary to neonatal events.^11^ The reality is different in developing countries, where, despite improvements in neonatal care and immunization programs, conditions related to the neonatal period and infections are still very common causes of hearing loss.^12^

Diagnostic evaluation of childhood hearing loss includes the investigation of patient history and physical examination, with audiology and electrophysiology testing. By performing additional investigation such as multidisciplinary evaluation, genetic and imaging tests,^8^ it is possible to identify the etiology in 50%–60% of all cases of hearing loss.^12^

The most important genetic tests are those that investigate the mutations of the Gap Junction Protein Beta 2 (GJB2) and Gap Junction Protein Beta 6 (GJB6) genes, which encode the 26 and 30 connexins, respectively. GJB2 mutations are the most prevalent, accounting for more than 50% of cases of non-syndromic genetic hearing loss. The 35delG mutation of the GJB2 gene is the most common.^1^ In cases with heterozygosis in GJB2, where there is no second mutation in the same gene that warrants the deafness phenotype, GJB6 investigation is recommended, since large GJB6 deletions are generally known to occur in combination with GJB2 recessive mutations.^13^, ^14^ The major mutations of GJB6 are del(GJB6-D13S1830) and del(GJB6-D13S1854).^13^

The identification of the main etiologies of childhood deafness is important for the primary prevention of this deficiency, through public health measures such as vaccination and improvement of maternal and child health. The early diagnosis of genetic deafness may also contribute to preventive measures, through genetic counseling.^5^, ^15^ Moreover, the miscegenation of the Brazilian population,^6^ with different ethnic groups in the various Brazilian regions, may suggest the existence of distinct genetic mutations amongst them. This reality makes it important to investigate the genetics of hearing loss in each region.

The primary objective of this study was to identify the most prevalent etiologies of hearing loss in a tertiary hospital in southern Brazil. Secondary objectives were to verify the prevalence of mutations in the genes of connexins 26 and 30 and to correlate the degree of hearing loss with the etiology of deafness.

## Methods

### Basic study settings and patient selection

This was a cross sectional study nested in a retrospective cohort in which patients from an outpatient clinic of a reference center for childhood deafness in tertiary hospital in southern Brazil were included. Referrals to this clinic occurred for two reasons: (a) failure in neonatal hearing screening (NHS) or (b) investigation and treatment of already diagnosed hearing loss.

Inclusion criteria for this study were as follows: (a) age up to 12 years incomplete; (b) bilateral sensorineural or mixed hearing loss; and (c) hearing loss that is congenital or acquired in the neonatal period. The project was approved by the Research Ethics Committee of the hospital where the research was conducted (number 150464). All adults responsible for the children signed the informed consent form.

### Patient assessment

At the first appointment of each patient, a medical history was gathered to elicit important information in order to characterize the child’s profile, the presence of risk factors for deafness and to hypothesize the etiological diagnosis of hearing loss. The assessed risk factors for hearing loss in childhood were the ones defined by the Joint Committee on Infant Hearing.^16^

Hearing loss acquired before birth, regardless of the time of manifestation of symptoms was considered congenital in this study. The term “conditions related to neonatal period” was used to describe neonatal conditions or complications that cause hearing loss and occur shortly after birth and may extend beyond the first month of life of the newborn. These conditions/complications are part of the risk factors determined by the Joint Committee on Infant Hearing, 2007.^16^

In order to demonstrate that the hearing loss was congenital or acquired in the neonatal period, the first criterion was the etiology of deafness. In case of undetermined etiology, we considered the result of the NHS. So, we assumed that patients with hearing loss without a defined etiology that had failed in the NHS were congenital.

### Audiological assessment

The type of hearing loss was defined by specific frequency auditory evoked potential and/or visual reinforcement audiometry (playful or tonal and vocal, according to age and ability of the child in answering the exam). The assessment of the degree of hearing loss was performed using the Interacoustics Eclipse EP25 ABR system® (Denmark) with NB-chirps® at 500 Hz, 1000 Hz, 2000 Hz and 4000 Hz or the Interacoustics AD 27 audiometer (with or without supra-aural phones TDH-3) and was classified according to the World Health Organization^17^ classification, which uses the mean square between auditory thresholds for frequencies of 500 Hz, 1000 Hz, 2000 Hz, and 4000 Hz. This classification divides hearing thresholds into mild (between 26 and 30 dB), moderate (between 31 and 60 dB), severe (between 61 and 80 dB) and profound (higher than 81 dB). Patients with secretory otitis media underwent tympanotomy for ventilation tube placement before definitive classification of type and degree of hearing loss.

### Laboratory and imaging tests

Laboratory tests to investigate congenital infections were carried out in the prenatal care of the pregnant women. In cases where the mother had not performed prenatal care, investigation was performed at the time of delivery and, depending on the result, also in the newborn, by means of serological tests, as recommended by the Brazilian Ministry of Health.^18^

Serology for cytomegalovirus (CMV) has also not been investigated, due to the low sensitivity and specificity of the presence of immunoglobulin M (IgM) in the diagnosis of congenital infection by this virus. Viral isolation in a culture of human fibroblasts or detection of viral DNA by polymerase chain reaction (PCR) in baby’s urine or saliva samples. This test was performed in symptomatic infants only.^19^

Patients with suspected inner ear malformation (craniofacial anomalies or syndromes, for example) or candidates for cochlear implants with severe or profound hearing loss underwent imaging examination: computed tomography (CT) and/or magnetic resonance imaging (MRI) of the ears. Patients with suspected syndromes, neurological or ophthalmologic disorders were referred for specialized evaluation.

### Genetic tests

Blood samples were collected from all non-syndromic children for DNA extraction and analysis of GJB2 gene mutations and deletion del(GJB6-D13S1830) of the GJB6 gene. Five mL of blood were collected in a tube with ethylenediaminetetraacetic acid (EDTA) and the material sent to the medical genetics laboratory to extract deoxyribonucleic acid (DNA) with the GENTRA Puregene Kit (Qiagen Inc., Valencia, USA).

For the genetic analysis of connexin 30, the samples were subjected to PCR for isolation of the fragment of interest, using three primers (1 forward and 2 reverse).^20^ The use of three primers in the same reaction allows evaluating the presence of the deletion del(GJB6-D13S1830) in the GJB6 gene, as well as whether it is present in heterozygosis or homozygous. The fragments were visualized by electrophoresis with 2% agarose gel. For the genetic analysis of connexin 26, the samples were also subjected to PCR for amplification. Then the amplified genetic materials were prepared and subjected to bidirectional sequencing by the method of Sanger.^21^

### Etiology groups

In order to facilitate the comparison of the data found with those of other studies, we grouped the etiological factors into six main groups:

1) Conditions Related to Neonatal Period (CRNP): Neonatal Intensive Care Unit (ICU) stay >5 days or extracorporeal ventilation, assisted ventilation, use of ototoxic drugs (gentamicin and tobramycin) or loop diuretics (furosemide) and hyperbilirubinemia requiring transfusion.^16^ We categorized all these factors into a single category for analysis purposes, because patients often have several of them concurrently. Patients were included in this group after the exclusion of the following etiological factors: congenital and neonatal infection, syndromic and non-syndromic genetic disorders, internal ear malformation and auditory neuropathy spectrum disorders.
2) Congenital or neonatal infection (CNI): infections acquired in the pre and perinatal period such as syphilis, toxoplasmosis, rubella, CMV, herpes virus, and acquired immunodeficiency virus (HIV).
3) Genetics: syndromic hearing loss (SHL) and non-syndromic hearing loss (NSHL). SHL consists of HL that presents with anomalies of the eye, kidney, the musculoskeletal and the nervous systems, as well as pigmentary disorders or others whereas, in our study, NSHL refers to patients with a genetic mutation in the GJB2 and/or GJB6 gene proven by molecular examination.
4) Auditory neuropathy spectrum disorder (ANSD): defined as the presence of otoacoustic emissions and/or cochlear microphonism and the absence of significant responses or changes in ABR.^22^, ^23^
5) Undetermined: after excluding other causes of hearing loss;
6) Others: malformations of the external, middle, or internal ear and central etiologies.

### Diagnostic steps groups

Another type of classification we performed was according to the steps of the investigative process of the etiology behind a hearing loss. The reason for that was to find how many patients can be diagnosed in one or two steps and how many needs more complex procedures.

The groups and criteria used to define each were as follows:

1) Medical history and physical examination: can identify conditions related to the neonatal period and congenital or neonatal infections, as well as most syndromes.
2) Auditory exams: can detect ANSD by means of suggestive findings in otoacoustic emissions and BERA.
3) Imaging exams: can detect the presence of inner ear malformations
4) Genetic tests: identifies the presence of mutations in the investigated genes that confirms if the hearing loss has non-syndromic genetic origin
5) Expert assessment: when patients with a set of suggestive changes of syndromes are referred for evaluation in the medical genetics service for the etiological diagnosis.

### Statistical analysis

A descriptive statistical analysis was performed. Quantitative variables were described by mean ± standard deviation and categorical variables, by total and absolute frequency. Pearson’s Chi-square test was used to compare the categorical variables. The SPSS 18.0 software was used for statistical analysis (SPSS Inc. released in 2009. PASW Statistics for Windows, version 18.0, Chicago: SPSS Inc.).

## Results

**Figure 1:**
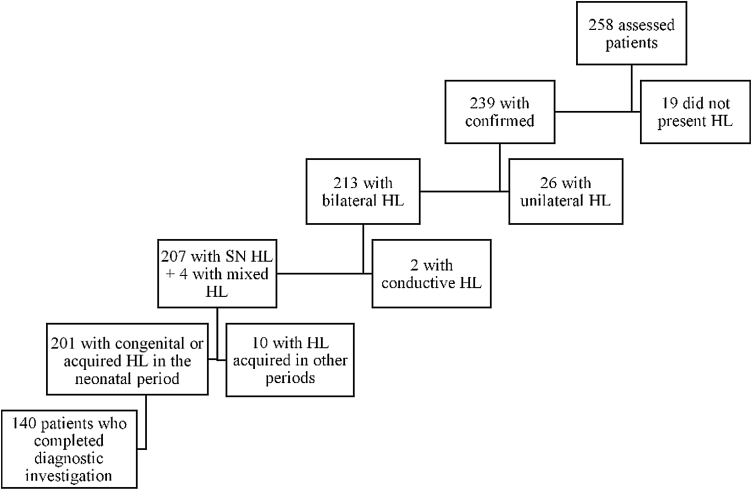
Organogram with the case series of the study. HL, hearing loss; SN, sensorineural.

In the period from January 2015 to December 2017, 258 children were referred to the outpatient clinic: 103 patients (39.9%) due to having failed the NHS and 155 patients (60.1%) for investigation and treatment of already diagnosed hearing loss. Deafness was confirmed in 239 (92,6%) patients, being bilateral in 213 (82,5%). Among these, 140 (54,2%) patients met the inclusion criteria, as shown by the organogram in Fig. 1.

### Patients profile

After the initial analysis, 140 children with a mean age (± standard deviation) at the first visit of 2.00-years (±2.29 years, 1–12 years) were evaluated. Seventy-two patients (51.4%) were male. As for race/color, 89.3% identified themselves as white, 7.9% black, 2.1% mixed, and 0.7% indigenous. Sixty-eight children (50.7%) were the children of multiparous mothers and 98.5% of the mothers performed prenatal consultations. Regarding the degree of hearing loss in the best ear, the mean was 96.43 dB (± 25.05).

Among evaluated children, 85 (60.7%) had at least one risk factor for childhood hearing loss.^16^ Twenty-seven children (19.3%) had a family history of hearing loss — nine had a sibling and eight had a parent with hearing loss. There were no reported cases of consanguinity.

### Etiology findings

**Figure 2:**
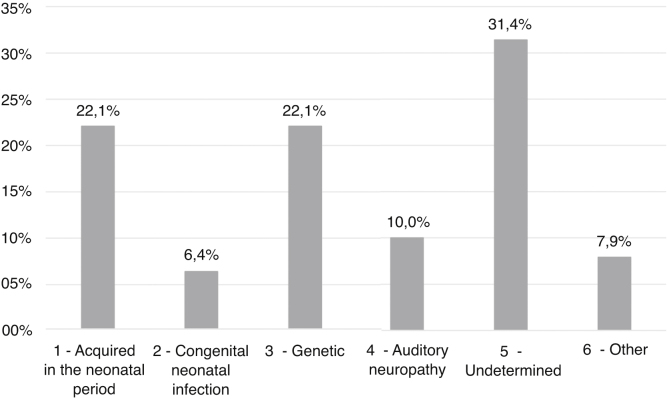
Main groups of hearing loss etiologies and their prevalence.

The most prevalent etiological groups were the ones of conditions related to neonatal period (Group 1) and genetics (Group 3), with 31 cases (22.1%) each. In 44 patients (31.4%) it was not possible to determine the cause of the hearing deficit (Group 5), as shown in Fig. 2.

In Group 1 (CRNP — Conditions Related to the Neonatal Period), neonatal ICU stay for more than five days was the most prevalent factor associated with hearing loss, occurring in 48 patients (34.3%), followed by mechanical ventilation (20.7%), ototoxic use (19.3%), and exchange transfusion for neonatal jaundice (6.4%).

CMV was the most prevalent infection of Group 2, with four cases (2.9%). We also identified two cases (1.4%) of congenital syphilis, 2 (1.4%) of toxoplasmosis and 1 (0.7%) with herpes virus infection. All these children were diagnosed with the congenital infection before the first consultation at the outpatient clinic. Seven children were symptomatic: four with CMV infection, two with toxoplasmosis and one with the herpes virus.

Hearing loss of genetic origin was divided into syndromic, 41.93%, and non-syndromic, 58.06%. Among the syndromic ones, we found two cases of CHARGE, one of Goldenhar, one of Waardenburg, one of Treacher Collins, one Stickler and seven cases of syndromic hearing loss, still without a definite diagnosis.

The genotypes of the 18 patients with non-syndromic genetic hearing loss and the number of cases of each are shown in Table 1.

**Table 1:** Genotype of non-syndromic genetic hearing loss.

Genotypes	n
35delG/35delG	10
35delG/N	5
35deG/p.Try172*	1
35delG/p.Arg184Pro	1
del(GJB6- D13S1830)/del(GJB6-D13S1830)	1
N/N	113

35delG/35delG, homozygous for 35delG; 35delG/N, heterozygous for 35delG; 35deG/p.Try172* and 35delG/p.Arg184Pro, heterozygous for 35delG with pathogenic variants of GJB2; N/N, without mutations.

Although genetic tests were performed on all non-syndromic children, regardless of any other possible etiology for hearing loss, only those with no other hypothesized etiology presented altered genetic tests. An important finding was that the final etiology in 44.44% of the children with a positive family history of hearing loss was genetic. Of those, 29.6% were non-syndromic (six homozygotes and two heterozygotes for the 35delG mutation) and 14.8% were syndromic (one CHARGE, one Waardenburg, one Treacher-Collins, and one in investigation, associated with peripheral facial nerve palsy).

Imaging exams (CT and/or MRI of the ears) were performed in 131 children (93.6%). Eight of these (6.1%) had inner ear malformations. Among them, we found a case of a single vestibulocochlear cavity, one of incomplete partition type 1, three of incomplete partition type 2, one of the vestibular enlarged aqueducts and two of cochlear nerve agenesis. Among the external and middle ear malformations: three cases of microtia and bilateral external auditory canal agenesis, one of them associated with middle ear malformation. All external ear malformations were found in syndromic patients (a case of Goldenhar, a case of Treacher- Collins and a syndrome under investigation), that is, they were not considered as the main etiologies. Also, from cases classified as central nervous system etiology, one was due to cortical malformation and two as sequelae of intracranial hemorrhage.

### Diagnostic steps findings

**Figure 3:**
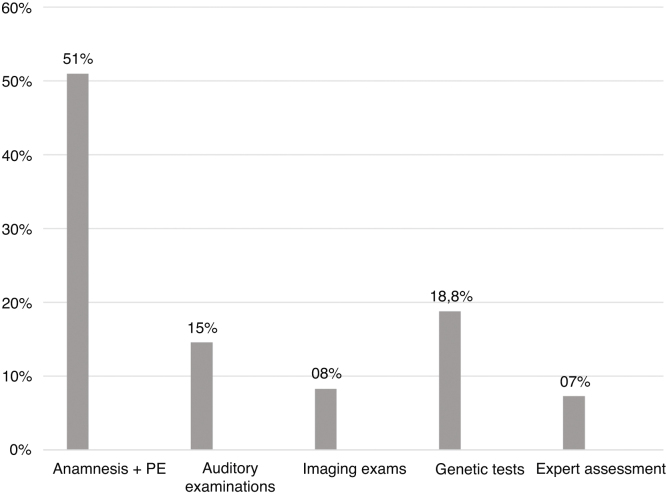
Investigation stages in which the diagnosis was defined. PE, physical examination.

Considering all the children with an established etiology for the hearing loss, 49 (51%) had the etiology established at the first appointment through medical history and physical examination. Genetic tests defined the cause in 18.8% of the cases. In Fig. 3, there are all the steps of the investigative process of the etiology behind a hearing loss, with the percentage of cases for which they were responsible.

### Audiological findings

**Figure 4:**
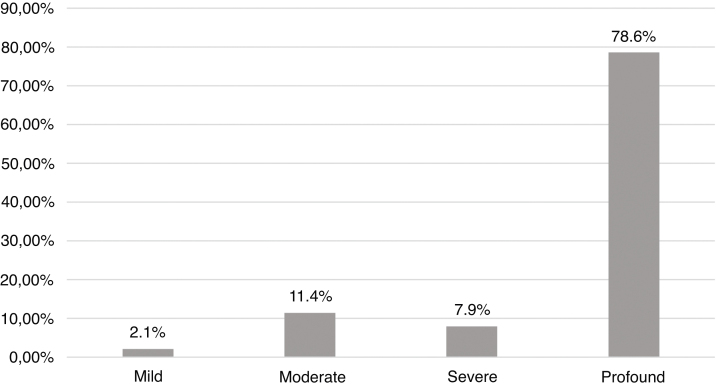
Classification of degrees of hearing loss in the best ear according to World Health Organization’s classification (2014).

Fig. 4 shows the distribution in degrees according to the World Health Organization classification (2014). In 78.6% the degree of hearing loss was profound. There was no statistically significant difference when comparing the degrees of hearing loss with the main etiologies.

## Discussion

According to this study, the two most prevalent etiologies for hearing loss were CRNP and CNI, which were responsible for a greater number of cases when compared with genetic etiology. Among NSHL groups, the 35delG mutation of the GJB2 gene was responsible for most cases.

It is difficult to compare our results with the ones from other studies. The differences in methods between ours and other studies are mainly in inclusion criteria (laterality, types, and degrees of hearing loss) and in classifications (of risk factors, etiology categories, degrees of hearing loss). However, because this is a prevalence study, results are expected to vary according to the genetic and socioeconomic characteristics of the analyzed populations and, since there are not similar studies conducted within the studied population, comparisons become less relevant. The results are therefore of great importance for the study of etiologies in different world scenarios.

The predominance of etiologies related to neonatal conditions and infectious causes are characteristic of developing countries, where investments on promotion and prevention of hearing health are limited.^24^ The frequency of these etiologies in other studies varies from 16% to 52%, with the lowest prevalence found in Finland, and the highest in sub-Saharan Africa.^3^, ^7^, ^25^, ^26^, ^27^ In this study, the prevalence was 28.5%, an intermediate value when compared to the world average and close to that found in other Brazilian studies.^26^, ^27^

Conditions related to the neonatal period (CRNP) were responsible for hearing loss of 22.1% of the 140 patients evaluated in a study by Walch et al. 19.8% of hearing loss was due to neonatal conditions^7^ while for Morzaria et al. it was 9.6%.^9^ That could be explained because in our sample there are many patients who were born in our hospital, which is a reference center for high-risk pregnancy in our state.

The prevalence of congenital infections was 6.4%, possibly reflecting improvements in hygiene methods, in prenatal and newborn care, as well as of vaccination campaigns. Rubella, which was once the most common viral cause of hearing loss,^12^ had a significant reduction after the introduction of vaccination.^9^ Brazil had the last registered case in 2009.^28^

In contrast to other congenital infections, CMV is being more studied in recent years, since it has become first place among the viral causes of deafness.^12^ In developed countries, it represents the second largest cause of hearing loss in children, with an incidence of 10%–12%.^3^, ^29^, ^30^ In our study, the majority of cases of infectious hearing loss were due to CMV all children identified with congenital CMV infection were symptomatic, since universal screening for CMV is not yet recommended by health authorities in Brazil.

With increasing control of neonatal conditions and infectious diseases plus the improved knowledge of deafness-related genes, genetic causes have become the main etiology of hearing loss in developed countries, making up to 50%–60% of cases. According to literature from across the globe, the prevalence varies from 15% to 60%.^3^, ^7^, ^9^, ^25^^,^^31^, ^32^ This variation can be explained by differences in ethnical and socio-economic development of the population analyzed, as well as by methodological variations between studies. In our sample, 22.1% of the cases were attributed to genetics, and the most prevalent mutations were analyzed, following recommendations regarding the investigation of congenital hearing loss.^19^, ^33^

NSHL was responsible for hearing loss in 12.9% of the children studied. Costa et al. also evaluated the causes of hearing loss in Brazilian children, and 16% of the cases were confirmed as genetic by molecular tests.^26^ Ethnicity has a significant influence on non-syndromic genetic hearing loss. GJB2 mutations, for example, are found predominantly in Europe, North Africa, the Middle East and in areas populated by immigrants from these locations.^1^, ^34^ The vast majority of participants in this study were self-declared Caucasian (89.3%), but it is well known that our population has important ethnic-racial mixtures. The characteristic miscegenation of Brazilian people began with South American Indians, Africans, and Portuguese colonizers. In the 19th and 20th centuries, immigrant flows entered Brazilian territory and in the southern region the immigration was mainly from Europe.^6^

Most of the mutations found in this study were in the GJB2 gene, which encodes connexin 26 (17 of the 18 children with alterations in molecular tests). The most prevalent mutation was 35delG, the main gene GJB2, ten mutations were found: three 35delG with biallelic expression (homozygous) and seven monoallelic (heterozygous).

Hearing loss occuring in homozygous patients and heterozygotes are called carriers^1^ and, according to the literature, about 1.35% to 6.5% of patients with hearing loss are carriers^1^, ^35^ with a high probability of the hearing loss being of genetic origin. Chang et al., described that 2.2% of the hearing-impaired population are carriers. According to him, the explanation lies in the existence of a GJB2 complementary gene that has not been identified, or of a carrier phenotype that is penetrating into a small fraction of GJB2 mutation carriers.^1^ For this reason, the five 35delG mutation cases of non-variant heterozygotes identified in this study (3.57% of all children) were considered as non-syndromic hearing loss cases.

Two other pathogenic variants of GJB2 were also found in heterozygous patients for 35delG. They were c.551G>C (p.Arg184Pro) and c.516G>A (p.Try172*). In this study, the p.Arg184Pro mutation was found in heterozygosis associated with the 35delG variant. This association has also been described in other studies.^36^, ^37^ The degree of hearing loss of the patient in this study was profound but Shalev et al. as well as Batissoco et al. described cases with moderate hearing loss.^25^, ^36^

The variant c.516G>A (p.Try172*) was described in the Brazilian population by Oliveira et al., through two cases of profound hearing loss with the p.Try172* in heterozygosis, one of them associated with 35delG.^38^ In this study, the same association and the same phenotype were identified. No other description of p.Try172* was found, suggesting that this variant may be typical of the Brazilian population.

The deletion del(GJB6-D13S1830), the most frequent of GJB6 mutations according to the literature, was found in a child in this study. These deletions remove all or part of the GJB6 gene, or regions neighboring it, and cause deafness when present in homozygosis or in heterozygosis in combination with a recessive mutation in the GJB2 gene. Although in most cases, they occur in combination with other GJB2 mutations, in this study, the patient was homozygous.^39^

Since the presence of risk factors would not exclude the possibility of concomitant genetic alteration, it was decided to perform the genetic tests in all non-syndromic children, independently of any other etiology being more likely. However, only those without a possible etiology presented alterations in the genetic tests. This result raises the argument about the need to perform genetic tests in all non-syndromic children.

Syndromic hearing loss was found in 9.3% of the total children in the study. Considering only the genetic etiology group, 41.93% were syndromic. According to the literature, prevalence is usually around 30%.^2^ The importance of this diagnosis is that it makes health care and patients aware of the prognosis of hearing loss and, especially, the associated complications, so that they can be properly managed. Of the 13 cases identified, seven had not yet been diagnosed, which is common. In many cases, it is difficult to identify the syndrome to which the set of different findings belongs. In addition, there are at least 45 genes associated with syndromic hearing loss, most of which have been discovered in the last two decades but are not available for testing in most clinical settings.^40^

The prevalence of children who remained without an etiological diagnosis was 31.4%. However, with the GJB2 and GJB6 mutations, 40% of nonsyndromic genetic hearing loss can be identified.^41^ If other genes causing hearing loss were investigated, we would probably decrease the number of children with no properly defined etiology. As an alternative, the use of multigenic panels to track a greater number of genes related to hearing loss is indicated. These panels have shown promising results in reducing costs and increasing the number of genes that can be screened simultaneously.^42^, ^43^

Congenital CMV infection is another possible cause of undiagnosed etiology for hearing loss. The explanation is probably related to the high prevalence of this infection and the difficulty of diagnosing the newborn because most are asymptomatic at birth and there is a short window of time to analyze CMV viral load in urine and/or saliva.^12^, ^29^ For this reason, universal screening for CMV in the maternity ward has been advocated. The early diagnosis of this congenital infection is very important because it is a potentially treatable cause of childhood hearing loss.^29^

About the steps of the investigative process of the etiology behind a hearing loss, it was already expected that the majority of cases could be identified through medical history and physical examination, since it was known that there is a high prevalence of hearing loss related to neonatal conditions. We observed that genetic tests were the second largest factor responsible for the definition of etiology (18.8%), which confirms the importance of the investigation of mutations in connexins 26 and 30 in cases of congenital hearing loss.

No impact of the different etiologies on the degree of hearing loss has been identified, which is in conformity with available literature data.^7^, ^22^, ^31^ However, the high prevalence of patients with profound loss may be a bias in our study since we are a reference center for cochlear implantation.

## Conclusion

The predominance of conditions related to neonatal period and infectious causes are characteristic of developing countries, and the high number of CRNP may also be due to our hospital being a reference center for high-risk pregnancy. The low prevalence of congenital infections possibly reflects improvements in hygiene methods, prenatal and newborn care, as well as of vaccination campaigns, but we believe that congenital CMV may play a bigger role than is currently known. The most prevalent mutation was 35delG, the main gene GJB2, probably because of the European influence in the genotype of our population. Fifty-one percent of the children with deafness had the etiology established at the first appointment, through medical history and physical examination. Genetic tests defined the cause in 18.8% of the cases.

## Conflicts of interest

The authors declare no conflicts of interest.
